# Drug-Coated Balloon Angioplasty for Dysfunctional Arteriovenous Hemodialysis Fistulae: A Randomized Controlled Trial

**DOI:** 10.2215/CJN.0000000000000359

**Published:** 2023-12-18

**Authors:** Yiping Zhao, Pei Wang, Yuzhu Wang, Lihong Zhang, Yu Zhao, Hua Li, Qiang He, Hao Liu, Jianfang Luo, Xin Jia, Zhengya Yu, Wei Guo, Lan Zhang

**Affiliations:** 1 Vascular Surgery Department, Renji Hospital, Shanghai Jiaotong University School of Medicine, Shanghai Jiaotong University, Shanghai, China; 2 Blood Purification Center, The First Affiliated Hospital of Zhengzhou University, Zhengzhou University, Zhengzhou, China; 3 Nephrology Department, Beijing Haidian Hospital, Beijing, China; 4 Nephrology Department, The First Hospital of Hebei Medical University, Hebei Medical University, Shijiazhuang, China; 5 Vascular Surgery Department, The First Affiliated Hospital of Chongqing Medical University, Chongqing Medical University, Chongqing, China; 6 Nephrology Department, Shaoyifu Hospital, Zhejiang Medical University, Hangzhou, China; 7 Nephrology Department, Zhejiang Provincial People's Hospital, Hangzhou, China; 8 Vascular and Interventional Radiology Department, Nanfang Hospital, Southern Medical University, Guangzhou, China; 9 Cardiology Department, Guangdong Provincial People's Hospital, Guangzhou, China; 10 Vascular Surgery Department, Chinese PLA General Hospital, Beijing, China; 11 Vascular Surgery Department, Tongren Hospital, Capital Medical University, Beijing, China

**Keywords:** arteriovenous fistula, chronic dialysis, chronic hemodialysis, clinical trial, dialysis access, hemodialysis access

## Abstract

**Background:**

The aim of this study was to evaluate the efficacy and safety of paclitaxel-coated balloons (AcoArt Orchid) in treating dysfunctional arteriovenous fistulae.

**Methods:**

The drug-eluting balloon for arteriovenous (AV) fistula in China trial was a prospective, multicenter, randomized controlled study. Patients who had ≥50% venous stenosis of the AV fistula and symptoms indicating significant hemodynamic changes were included. After successful predilation with a high-pressure balloon (residual stenosis ≤30%), patients were randomized 1:1 to either a paclitaxel-coated balloon or an uncoated control balloon. The primary efficacy outcome was assessed at 6 months, and safety assessment was conducted within 30 days of the procedure. The 12-month results were also analyzed.

**Results:**

The study included 244 patients, equally distributed between the two groups. The primary target lesion patency was 91% (106/116) for the drug-coated balloon (DCB) group and 67% (79/118) for the plain balloon catheter group, representing a difference of 24.63% (95% confidence interval, 14.68 to 34.58; *P* < 0.001). The secondary efficacy end point was primary target lesion patency at 12 months, which was 66% (74/112) for the DCB group and 46% (52/112) for the plain balloon catheter group (95% confidence interval, 6.57 to 32.08; *P* = 0.004). The mean number of reinterventions per patient to maintain target lesion patency during the 12 months after the index procedure was 0.39 (48/122) in the DCB group and 0.77 (94/122) in the plain balloon catheter group (*P* = 0.001). The primary safety end point did not differ between groups (*P* = 0.25).

**Conclusions:**

AcoArt Orchid DCB showed better primary patency rates compared with plain balloon angioplasty for treating stenotic lesions in dysfunctional hemodialysis AV fistulae at 6 and 12 months. It required fewer repeated interventions and had comparable safety in 1 year.

**Clinical Trial registry name and registration number:**

AcoArt III/Arterio-venous Fistula in China, NCT03366727.

## Visual Abstract

**Figure:**
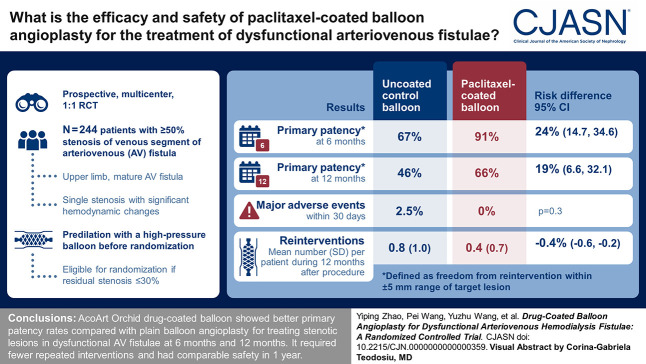

## Introduction

Kidney failure can be a significant burden on health care systems, requiring either kidney transplant or long-term dialysis therapy. A previous study conducted in 2017 found that there were 697.5 million cases of CKD worldwide, resulting in a global prevalence of 9.1%; notably, China and India account for almost one third of these cases.^1^ For individuals with kidney failure, the number of people requiring KRT currently exceeds 2.5 million, and this is projected to increase to 5.4 million by 2030.^2^ Hemodialysis is the primary modality of KRT, with 91.0% of patients with kidney failure receiving hemodialysis.^3^ In China, arteriovenous (AV) fistula is the most prevalent type of vascular access for hemodialysis, accounting for over 80% of cases.^4^

AV fistula dysfunction caused by vascular stenosis is a major issue with the widespread adoption of autologous AV fistulae, while endovascular angioplasty has proven to be an effective treatment for stenoses in arteriovenous access.^5–7^ Although the mechanism of arteriovenous access stenosis is complex and not entirely understood, intimal hyperplasia is considered one of the primary causes. Therefore, an effective solution to inhibit intimal hyperplasia could help reduce the rate of restenosis and maximize patency time. The use of paclitaxel drug-coated balloons (DCBs) has been proven to be effective in reducing neointimal hyperplasia and preventing vascular restenosis in various arteries, including coronary and femoral arteries.^8–12^

The IN.PACT trial demonstrated that the use of IN.PACT DCB resulted in significantly higher target lesion primary patency compared with standard balloons.^13^ In the APERTO AVF RCT China trial, a study comparing APERTO DCB (a new high-pressure DCB) with a noncoated balloon, the freedom from a clinically driven target lesion revascularization rate at 6 months was 82.0% for the DCB group versus 75.0% for the noncoated balloon group, and 73.0% versus 58.0% at 12 months.^14^

The drug-eluting balloon for AV fistula study, which was a multicenter, randomized, controlled trial, aimed to assess the safety and efficacy of AcoArt Orchid, a paclitaxel-coated balloon, in treating dysfunctional arteriovenous fistulae. This DCB has already demonstrated promising results in preventing restenosis in femoral arteries and has been used in the treatment of femoral popliteal artery.^9–11^ As the first domestic DCB in China, the success of this treatment could lead to longer periods of successful and uninterrupted dialysis for patients receiving hemodialysis, thereby significantly improving the treatment status of patients with kidney failure in China.

## Methods

### Trial Design

The drug-eluting balloon for AV fistula trial is a prospective, multicenter, 1:1 randomized clinical trial aimed at demonstrating the safety and efficacy of the AcoArt Orchid DCB for treating *de novo* or restenotic lesions in dysfunctional arteriovenous fistulae. The patients enrolled might have undergone previous angioplasty using a plain old balloon before trial enrollment. The trial was conducted at 11 tertiary centers throughout China and was performed under the US Food and Drug Administration investigational device exemption in compliance with the country's laws and regulations. The study was also performed in accordance with the Declaration of Helsinki. The trial protocol, including amendments, is available at ClinicalTrials.gov (NCT03366727) and was reviewed and approved by the ethics committee or institutional review board at each site.

The main inclusion criteria for this study are as follows: (*1*) The patient's AV fistula must be matured and have undergone at least one successful hemodialysis procedure. (*2*) The target lesion must be located in the venous segment of the AV fistula in the upper limb, excluding lesions in the artery, anastomosis, and central vein. (*3*) The length of the target lesion must not exceed 100 mm. (*4*) The residual stenosis after predilation must be ≤30%. (*5*) The degree of target lesion stenosis must be at least 50% as determined by angiography and exhibit one of the symptoms such as significantly increased venous pressure during dialysis, abnormal physical examination, or significantly decreased blood flow (<200 ml/min).

The main exclusion criteria for this study are as follows: (*1*) Patients with more than two target lesions. However, if the two stenosis lesions are closer than 3 cm, they are considered as one lesion. (*2*) AV fistula located at lower limbs. (*3*) In-stent restenosis. (*4*) AV fistula with acute thrombosis that received thrombolysis or thrombectomy in the past 30 days, or requires these treatments in the index procedure. (*5*) AV fistula that received open surgery in the past 30 days or is intended to undergo surgery. (*6*) AV fistula infection or systemic active infection.

As to the randomization method, eligible patients were randomly assigned in a 1:1 ratio to the different treatment groups using a central randomization system (Interactive Web Response System). The randomization was not stratified for site or clinical characteristics. As to the masking design, owing to the visual characteristics of medical devices, achieving double blinding was challenging. Therefore, single blinding could only be implemented from the perspective of the patients. This meant that patients were unaware of whether they were in the experimental or control group, to prevent subjective assumptions and other factors that might interfere with the assessment of effectiveness. The people who performed the procedure was not involved in data collection. As to the blind evaluation of end point events, all recorded data regarding end point events, collected by the researcher in the case report form, were submitted to an independent third-party Clinical Events Committee (CEC) for evaluation. This meant that the CEC members did not know whether the patient was in the experimental or control group. Only those events that were deemed positive by the CEC were included in the statistical dataset for the primary end point indicator. The clinical monitoring process for this study involved a thorough 100% retraceable source document verification, and any adverse events that occurred were duly reported to the Beijing Medical Products Administration. The Medical Research and Biometrics Center (National Center for Cardiovascular Diseases, Beijing, China) was responsible for data management and statistical analysis.

Acotec Scientific Ltd., sponsored this study, which was conducted in accordance with the Declaration of Helsinki and the guidelines for conducting clinical trials of medical devices issued by the National Medical Products Administration. The ethics committees of all participating hospitals reviewed and approved the study, and all patients provided informed consent before undergoing any study procedures.

### Study Device

The AcoArt Orchid DCB is a DCB that contains a paclitaxel dose of 3.3 *μ*g/mm^2^, along with a magnesium stearate excipient. This 0.035-in guidewire-compatible device is available in a range of sizes, including balloon diameters of 4–12 mm, and two effective rod lengths of 80 and 130 cm, which were used in the trial. The control device used in the study was the Admiral Xtreme Plain Balloon Catheter (Medtronic).

### Study Procedure

After meeting the National Kidney Foundation Kidney Disease Outcomes Quality Initiative Guidelines,^15^ eligible participants underwent predilation with a high-pressure balloon to ensure initial medical performance standards were met. The diameter of the balloon was selected based on vessel diameter (1:1 sizing) as determined by digital subtraction angiography. The recommended inflation time was 2 minutes, at the discretion of the interventionalists. Successful predilation was achieved when the residual stenosis was no more than 30% of the vessel diameter, determined through visual estimation by the interventionalists. Only after successful predilation were participants enrolled and randomly assigned to treatment with either a DCB or a plain old balloon (not a high-pressure balloon) in a 1:1 ratio. In both groups, the diameter of the study balloon remained identical to the predilation balloon. However, in the DCB group, the length of the balloon was extended beyond the predilation balloon (by at least 5 mm) to avoid geographic miss.

### End Points and Definitions

The primary end point of this study was the primary patency of the target lesion after 6 months, which was defined as freedom from reintervention within a range of ±5 mm of the target lesion. Reintervention of the target lesion was necessary when there was clinically driven target lesion reintervention, thrombosis, surgical removal of the target lesion, or abandonment of AV fistula because of inability to repair the target lesion.

The key secondary end points included the primary patency of the target lesion after 12 months, the number of reinterventions required to maintain target lesion patency in 12 months after the index procedure. Other secondary end points included device success, which was defined as the successful delivery, inflation, and retrieval of the device without rupture during the procedure, evaluated on a single device. Clinical success was defined as the successful completion of hemodialysis more than once after the index procedure. Procedural success was defined as residual stenosis of the target lesion ≤30% by visual estimation, without the occurrence of major adverse events during the perioperative period. The device success and procedural success were evaluated immediately postprocedure by physicians, whereas clinical success was evaluated by investigators 0–5 days after the procedure. The clinicians who determined the need for clinically driven target lesion reintervention were blinded to randomization assignment.

The safety end point was defined as major adverse events, including all-cause death, stroke, and pulmonary embolism within 30 days after the index procedure. Preoperation angiographic findings were evaluated by an independent third party, the CEC, whose positive events were used to calculate primary patency.

### Follow-Up

All patients were followed up for a minimum of 12 months, with all follow-up visits being clinically driven and not scheduled for angiography or ultrasonography. Efficacy outcomes were assessed at 3, 6, 9, and 12 months through outpatient services or telephone visits. Any unscheduled visits were carefully recorded throughout the trial, particularly those that necessitated repeat interventions.

### Statistical Analysis

According to the results of a previous study, the rate of target lesion primary patency over 6 months in the plain balloon catheter group was around 60%, whereas the rate in the DCB group was approximately 80%.^13^ Patients were randomly assigned in a 1:1 ratio to either the DCB or the plain balloon catheter group. With a significance level (*α*) of 0.05, power of 80%, and 15% expected dropout rate, a total of 122 patients were required for both groups. The primary analysis utilized an intention-to-treat approach. Comparisons between groups were conducted using the Cochran–Mantel–Haenszel test, with the center effect taken into account. The risk difference was used as the effect size for binary outcomes, such as target lesion primary patency over 6 or 12 months. In addition, considering clinical center as a random effect, the generalized estimation equation model was used to compare the difference between groups. Time-to-event analyses were also performed using the Kaplan–Meier method. A competing risk model was utilized when counting the reintervention risk, considering death as a competing event.^16^ The Wilcoxon signed-rank test was used to compare the number of reinterventions between two groups within 12 months. The differences of device success, clinical success, and procedural success between two groups were compared using the Fisher exact test. For the safety end point, between-group differences were assessed using the chi-square test or Fisher exact test. Prespecified sensitivity analyses, including multiple imputation with logistic regression (fully conditional method) and worst-case carry-forward imputation, were conducted to assess the effect of missing data on the primary outcome. The Glimmix procedure in SAS 9.4 was used to perform the subgroup analysis for treatment by subgroup interactions. Subgroup analyses were planned for baseline characteristics, including age, sex, handedness, medical history (including hypertension, diabetes, and hyperlipidemia), number of lesions, target vascular surgery, and months of hemodialysis. All statistical analyses were performed at a significance level of 0.05, and SAS 9.4 was used for the analyses. Forest plots were generated using an R package forester.^17^

## Results

### Baseline Characteristics

**Figure 1:**
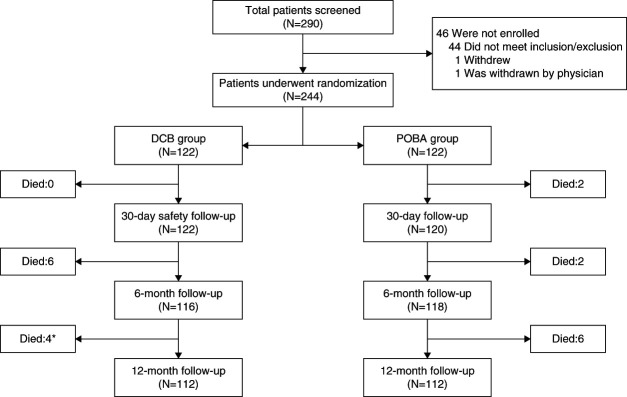
**Flow diagram of patients.** *One participant died of suicide. DCB, drug-coated balloon; POBA, plain old balloon angioplasty.

Between January 2017 and December 2021, a total of 244 patients were enrolled across 11 medical centers in China and assigned to either the DCB or the plain balloon catheter treatment group in a 1:1 ratio (as depicted in Figure 1). There were no significant differences in demographic baseline or target lesion characteristics between the two groups, as summarized in Table 1. Procedural characteristics were generally comparable between the groups, with the exception of the study balloon diameter, length, and inflation time (as outlined in Supplemental Table 1). Specifically, the diameter of the experimental balloon was found to be larger in the plain old balloon angioplasty group than in the DCB group (6.1±0.7 versus 5.9±0.8 mm, *P* = 0.03), whereas the experimental balloon was longer in the DCB group than in the plain old balloon angioplasty group (68.3±12.5 versus 50.4±10.0 mm, *P* < 0.001). In addition, the inflation time for the experimental balloon was found to be longer in the DCB group than in the plain old balloon angioplasty group (126±20 versus 108±33 seconds, *P* < 0.001). The median follow-up time in the DCB group was 354 days (25th percentile: 348 days, 75th percentile: 362 days) and that for the plain old balloon angioplasty group was 357 days (25th percentile: 348 days, 75th percentile: 366 days). There was no significant difference in the follow-up time between two groups (*Z* value: 1.196, *P*: 0.2316).

**Table 1:** Patient demographic and clinical characteristics at baseline

Characteristics	DCB (*N*=122)	PTA (*N*=122)	Overall (*N*=244)
Age, yr	57±13	58±14	58±13
Male sex, no. (%)	75 (62)	69 (57)	144 (59)
**Causes of hemodialysis, no. (%)**			
Primary glomerular disease	10 (8)	12 (10)	22 (9)
Chronic interstitial nephritis	3 (3)	5 (4)	8 (3)
Diabetic kidney disease	31 (25)	27 (22)	58 (24)
Hypertensive kidney disease	11 (9)	11 (9)	22 (9)
Kidney calculus	1 (1)	1 (1)	2 (1)
Polycystic kidney disease	7 (6)	7 (6)	14 (6)
Unknown	17 (14)	14 (12)	31 (13)
Others	69 (57)	68 (56)	137 (56)
**Target AV fistula, no. (%)**			
Fistula vintage, mo (mean±SD)	31.8±9.2	26.0±8.9	28.9±9.1
Left arm	85 (70)	92 (75)	177 (73)
On dominant arm	37 (30)	31 (25)	68 (28)
On upper arm	3 (3)	3 (3)	6 (3)
End-to-end anastomotic mode	8 (7)	7 (6)	15 (6)
Anastomotic vein (cephalic vein)	119 (98)	119 (98)	238 (98)
Anastomotic artery (radial artery)	116 (95)	117 (96)	233 (96)
Usage time before index procedure, mo, (mean±SD)	31.8±33.4	26.0±30.0	28.9±31.8
**No. of target lesion per patients**			
1	106 (87)	108 (89)	214 (88)
2	16 (13)	14 (12)	30 (12)
**Has previous treatment history of target AV fistula**	85 (70)	84 (69)	169 (69)
PTA group	82 (97)	75 (89)	157 (93)
Surgery	3 (4)	9 (11)	12 (7)
**Medical conditions, no. (%)**			
Hypertension	104 (85)	106 (87)	210 (86)
Hyperlipidemia	24 (20)	21 (17)	45 (18)
Diabetes mellitus	44 (36)	54 (44)	98 (40)
Coronary heart disease	26 (21)	29 (24)	55 (23)
Peripheral artery disease	4 (3)	2 (2)	6 (33)
Cancer	4 (3)	7 (6)	11 (5)

AV, arteriovenous; DCB, drug-coated balloon; PTA, percutaneous transluminal angioplasty using plain old balloon.

### Efficacy Analyses

The study found that the DCB group had a significantly higher percentage of participants with target lesion primary patency during the 6 months after the index procedure compared with the percutaneous transluminal angioplasty group. Specifically, target lesion primary patency was achieved in 91% (106 of 116) in the DCB group and 66.95% (79 of 118) in the plain balloon catheter group, resulting in a risk difference of 24.63 percentage points (95% confidence interval [CI], 14.7 to 34.6; *P* < 0.001; as provided in Table 2). The result of the generalized estimation equation model also suggested that the relative risk value for the DCB group was 1.37 with a 95% CI of 1.19 to 1.57 when compared with the control group. As to the sensitivity analyses, the results of multiple imputation and worst-case carry-forward imputation confirmed the significant risk difference between two groups (*P* < 0.0001, Table 2).

**Table 2:** Primary and key secondary end point

End Point	DCB Group	PTA Group	Risk Difference, % (95% CI)	*P* Value
**Primary effectiveness end point: target lesion primary patency over 6 mo (patient level)**				
Primary analysis, no./total no. (%)	106/116 (91)	79/118 (67)	24.6 (14.7 to 34.6)	<0.001
Multiple imputation	—	—	22.5 (12.1 to 32.9)	<0.001
Worst-case analysis, no./total no. (%)	106/122 (87)	79/122 (65)	22.0 (11.5 to 32.5)	<0.001
**Key secondary end points**				
Target lesion primary patency over 12 mo, no./total no. (%)	74/112 (66)	52/112 (46)	19.32 (6.6 to 32.1)	0.004
No. of reinterventions within 12 mo, no. (%)	48 (1)	94 (1)	−0.4 (−0.6 to −0.2)	<0.001
Target lesion primary patency over 6 mo (lesion level), no./total no. (%)	120/132 (91)	92/132 (70)	21.3 (11.9 to 30.7)	<0.001

CI, confidence interval; DCB, drug-coated balloon; PTA, percutaneous transluminal angioplasty using a plain old balloon.

**Figure 2:**
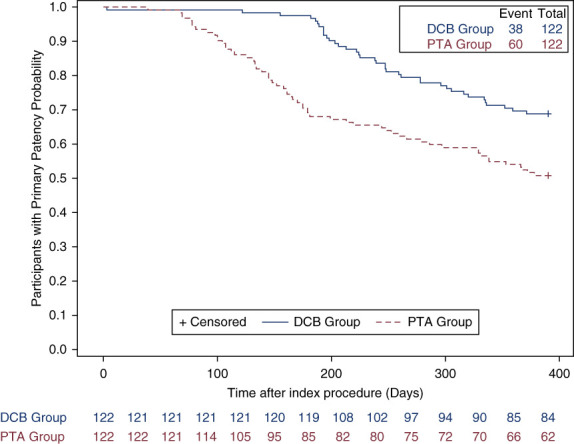
**Kaplan–Meier analyses of primary patency in 12 months after the index procedure.** As the 6-month target lesion primary patency was the primary end point, the difference between the two curves was significant at 6 months (logrank *χ*²=11.12, *P* = 0.0009). PTA, percutaneous transluminal angioplasty using a plain old balloon.

To illustrate the time course of primary outcomes, the Kaplan–Meier analysis showed that the cumulative target lesion primary patency of the DCB group was significantly higher than that of the plain balloon catheter group at 92.3% and 66.9%, respectively (Figure 2). Even when considering patients with two target lesions, the target lesion primary patency at the lesion level during the 6 months remained statistically different (90.91% [120 of 132] versus 69.7% [92 of 132], risk difference, 21.3 percentage points; 95% CI, 11.91 to 30.69; *P* < 0.001; as given in Table 2).

At 12 months, the target lesion primary patency remained statistically different between the two groups (66.1% [74 of 112] versus 46.4% [52 of 112], risk difference, 19.32 percentage points; 95% CI, 6.57 to 32.08; *P* = 0.0037; as given in Table 2). The Kaplan–Meier analysis also showed that the freedom from reintervention was significantly higher in the DCB group at 67.5% compared with 48.6% in the plain balloon catheter group at 12 months (*P* = 0.0006, as shown in Figure 2).

The number of interventions required to maintain target lesion patency was also significantly lower in the DCB group, with 48 interventions compared with 94 in the plain balloon catheter group. The mean number of reinterventions per patient during the 12 months after the index procedure was 0.4 in the DCB group and 0.8 in the plain balloon catheter group (*P* = 0.001, Table 2 and Supplemental Figure 1).

### Subgroup Analysis of the Primary and Secondary End Points

**Figure 3:**
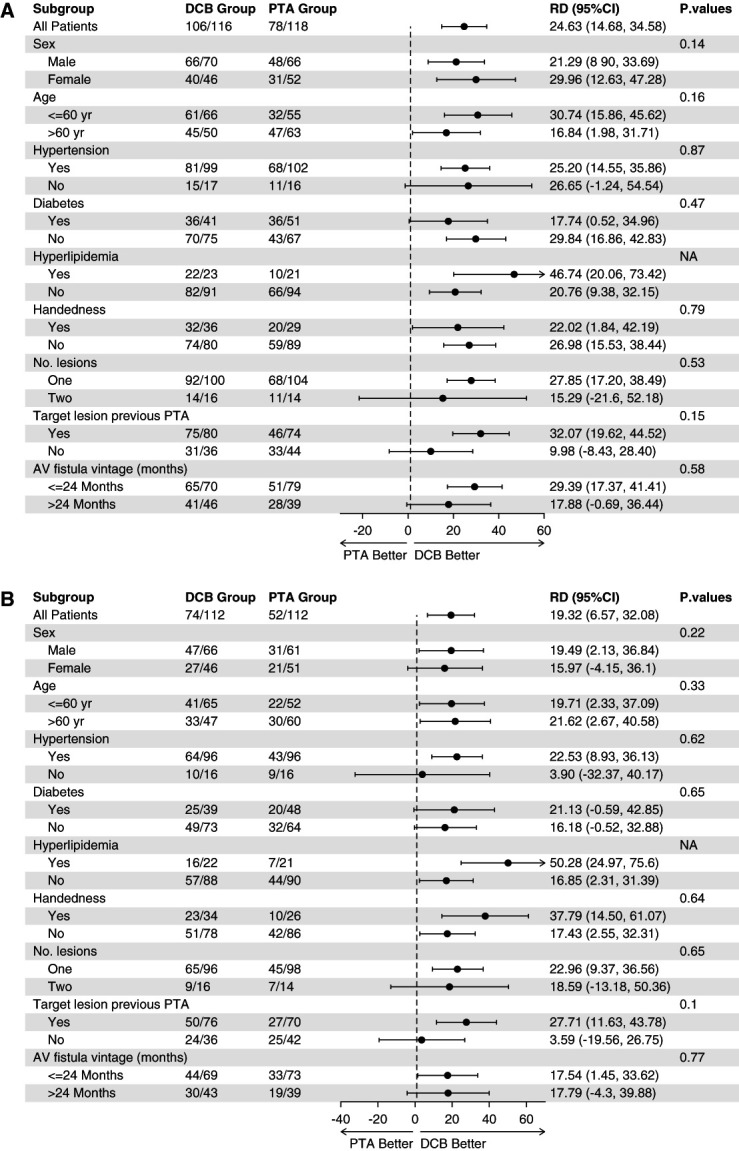
**Subgroup analysis of the primary and secondary end points.** Subgroup analysis for the outcome of primary patency of target lesions at 6 months (A) and 12 months (B). *P* values represent interaction tests. AV, arteriovenous; CI, confidence interval; RD, risk difference.

The subgroup analyses were conducted in sex, age, hypertension, diabetes, hyperlipidemia, handedness, number of lesions, target lesion previous percutaneous transluminal angioplasty, and AV fistula vintage. These were analyzed in 6 and 12 months (Figure 3). After the interaction among subgroup variables were considered, the efficacy of DCB did not differ in these subgroups (Supplemental Table 2).

### Safety End Points

When analyzing the percentage of participants experiencing major adverse events, such as all-cause death, stroke, and pulmonary embolism, within 30 days postprocedure, the results indicated that there was no significant difference between DCB group and plain balloon catheter group (0% [0 of 122] versus 2.5% [3 of 122], *P* = 0.3, as given in Table 3). In 12-month follow-up, there were 231 adverse events in the DCB group and 256 in the control group. Sixty-nine patients in the DCB group experienced adverse events, whereas 79 patients in the control group. The incidence difference was not significant (56.56% [69 of 122] versus 64.75% [79 of 122], *P* = 0.2, as provided in Table 3). There was one endo-device–related adverse event recorded in the control group, but the incidence was insignificant between two groups.

**Table 3:** Safety end point

Safety End Point	DCB Group	PTA Group	*P* Value
**Within 30 d**			
Major adverse events, %	0%	3% (3/122)	0.3
*Death*	0%	2% (2/122)	0.5
*Stroke*	0%	2% (2/122)	0.5
*Pulmonary embolism*	0%	0%	1.0
**Within 12 mo**			
The no. of patients with adverse events^a^	57% (69/122)	65% (79/122)	0.2
*Endo-device–related adverse events*	0%	1% (1/122)	1.0

DCB, drug-coated balloon; PTA, percutaneous transluminal angioplasty using a plain old balloon.

a The numbers were patients who experienced one or more than one adverse event.

## Discussion

Although autologous fistula is becoming the primary option for patients undergoing hemodialysis, it is prone to developing stenoses due to the circuit's physiologic nature, often resulting in intervention to restore function. Regrettably, approximately 50% of patients require repeat endovascular therapy within 6 months.^5–7,18^ Over the past 50 years, various treatments for fistula stenosis have been introduced, such as percutaneous transluminal angioplasty, cutting balloons, and stent grafts, among others. Although some of these treatments have shown potential, the combined treatment modalities for fistulas did not demonstrate a significant advantage over conventional angioplasty.

This study builds on smaller, randomized, single-center studies that showed benefit of the DCB over angioplasty.^19–23^ Previous studies have shown potential benefits of DCB angioplasty and prolonged survival time compared with conventional high-pressure balloon angioplasty.^18,20,24^ Our study, which involved 244 participants, builds on the positive findings of previous research. We found that DCB treatment was more effective than conventional angioplasty in improving the patency rate, indicating that DCBs are superior in maintaining the results of angioplasty after 6 and 12 months. This marks the first ever clinical trial of a domestically produced DCB in China, and our results suggest that its efficacy is comparable to that of other trials. Specifically, we saw a 24.4% improvement in target lesion primary patency between DCB and plain old balloon angioplasty using a plain balloon catheter after 6 months, and a 19.7% improvement after 12 month. These figures were also consistent with those reported in the IN.PACT trial, which saw a 22.7% improvement after 6 months and 19.0% improvement after 12 months.^13^ Compared with previous studies that failed to achieve their primary end point or produced conflicting results, this study yielded favorable outcomes. The success of this study may be attributed to the protocol that required adequate percutaneous transluminal angioplasty before DCBs. Only participants who underwent successful predilation using a high-pressure plain balloon catheter, resulting in residual stenosis that did not exceed 30% of the vessel diameter, were enrolled and randomly assigned to two different treatment groups. This approach effectively eliminated confounding factors caused by insufficient predilatation and accurately tested the efficacy of DCBs in preventing dialysis access restenosis. This experience highlights the crucial role of successful predilation, which is consistent with findings from DCB trials in the arteriosclerotic lesions of the femoral artery.^9,10^ The mechanism behind inadequate predilation leading to significant restenosis may be related to the hemodynamic effect of residual stenosis on intimal hyperplasia.^10^

A subset analysis was conducted to identify the subgroups that benefited more from DCBs. On consideration of the interaction of the subgroup, we found that there was no significant difference between the two groups in these subgroups. Previous studies did not indicate a positive subset analysis.^25^ However, as more studies are conducted, the data will allow for more subset analyses, revealing more specific details about the subgroup patients who may benefit from DCBs. The relatively small subgroup size might have contributed to the underpowered analyses.

The study has successfully achieved its primary safety outcome, indicating that paclitaxel, in the specific dosage and delivery method utilized, does not exhibit any toxicity. Although there is no evidence to suggest the occurrence of late complications related to the DCB, it is possible that hemodialysis access may require frequent use of the balloon because of the aggressive nature of restenosis in this setting compared with arterial disease. However, even with repeated usage in small doses, the likelihood of long-term adverse effects is highly unlikely.

The most objective and standardized clinical monitoring parameter for fistula flow would have been flow measurement. However, as flow measurement was not available in all centers, it was not required in this study. Although the use of the blinded core laboratory strengthens the observations and measurements, there may have been variability in the quality of angioplasty and/or drug delivery among investigators. Moreover, to ensure the full coverage of the target lesions with paclitaxel, in the DCB group, the length of the balloon was extended beyond the predilation balloon, and this procedure might have contributed to the superiority of the DCB cohort. In addition, the DCB balloon was not just the control balloon with paclitaxel added but was structurally different and the duration of inflation was longer in the DCB cohort, which may also contribute to the differences in the outcome. Ideally, the study could have been double-blinded, but the trial device had a different appearance from a standard angioplasty balloon, which made it impossible to blind the investigator performing the procedure. Nevertheless, blinding the patient and dialysis center to the treatment performed is believed to have substantially mitigated this issue. The subgroups in our study were relatively small, which might have limited ability to detect significant differences in subgroup analyses.

The trial the drug-eluting balloon for AV fistula in China demonstrated that, after successful predilation, DCB angioplasty using the AcoArt Orchid DCB was superior to standard angioplasty in treating stenotic lesions in dysfunctional hemodialysis AV fistulae at both 6- and 12-month time points. Moreover, it was found to be noninferior regarding major adverse events at both 30 days and 12 months.

## Supplementary Material

SUPPLEMENTARY MATERIAL

## Data Availability

All data are included in the manuscript and/or supporting information.

## Supplemental Material

Supplemental Table 1. Procedural data of the target AV fistula between two groups.

Supplemental Table 2. The reintervention numbers in 6 and 12 months.

Supplemental Figure 1. Cumulative incidence functions of reintervention risk during 12 months.
