# Supplementary material for: Drug-Coated Balloon Angioplasty for Dysfunctional Arteriovenous Hemodialysis Fistulae: A Randomized Controlled Trial
Source: Clin J Am Soc Nephrol. 2023 Dec 18;19(3):336–44. doi: 10.2215/CJN.0000000000000359 (PMC10937027; doi:10.2215/CJN.0000000000000359)
Supplement: SUPPLEMENTARY MATERIAL [file cjasn-19-336-s001.pdf]

Supplemental Material

Supplemental Table 1. Procedural data of the target AVF between two groups

Supplemental Table 2. The reintervention numbers in 6 months and 12 months

Supplemental Figure 1. Cumulative incidence functions of reintervention risk during 12 months

Supplemental Table 1. Procedural data of the target AVF between two groups.

Characteristics	DCB (N=122)	PTA (N=122)	Overall (N=244)	P value
Intervention access				0.7
Arterial antegrade access	13(10.8)	18(14.8)	31(12.7)	
Venous antegrade access	10(8.2)	11(9.0)	21(8.6)	
Venous retrograde access	95(77.9)	87(71.3)	182(74.6)	
others	4(3.3)	6(4.9)	10(4.1)	
AVF anastomosis lesion	2(1.6)	2(1.6)	4(1.6)	1
Distance between lesion and radiocarpal joint (cm)	18.5±17.9	22.2±222.0	20.4±20.1	0.2
Length of target lesion (cm)	33.1(20.9)	35.3(22.4)	34.2(21.6)	0.4
Diameter of reference vessel# (mm)	5.8(0.8)	5.9(0.9)	5.8(0.8)	0.2
Stenosis rate before treatment	79.9±10.3	79.4±12.1	79.7±11.2	0.7
Number of pre-dilatation balloon				
1	118(85.5)	119(87.5)	237(86.5)	0.9
2	19(13.8)	17(12.5)	36(13.1)	
3	1(0.7)	0(0.0)	1(0.4)	
Final diameter	5.8 (0.99)	5.9 (0.9)	5.8 (0.9)	0.1

of pre-dilatation balloon (mm)				
Final length of pre-dilatation balloon (mm)	47.3±10.4)	46.4±10.7	46.9±10.5	0.5
Diameter of the experimental balloon (mm)	5.9±0.8	6.1±0.7	6.0±0.8	0.0
Length of the experimental balloon (mm)	68.3±12.5	50.4±10.0	60.5±14.5	<0.001
Time of experimental balloon inflation (seconds)	125.9±19.8	107.7±32.9	118.0±27.7	<0.001
Pressure of experimental balloon inflation (atm)	12.2±2.6	12.6±3.2	12.4±2.9	0.3

DCB: drug-coated balloon. **PTA:** Percutaneous Transluminal Angioplasty. #: reference vessel referred to the non-stenotic venous segment of the AV fistula which was close to the target lesion. It was used to choose the diameter of the balloon.

Supplemental Table 2. The reintervention numbers in 6 months and 12 months

Group	PTA	thrombolysis	Surgery removal of AVF	AVF abandonment	Total count
6 months					
DCB Group	9	0	0	1	10
PTA Group	42	0	1	2	45
12 months					
DCB Group	45	1	1	1	48
PTA Group	90	0	1	3	94

DCB: drug-coated balloon. PTA: Percutaneous Transluminal Angioplasty. AVF: Arteriovenous fistula.

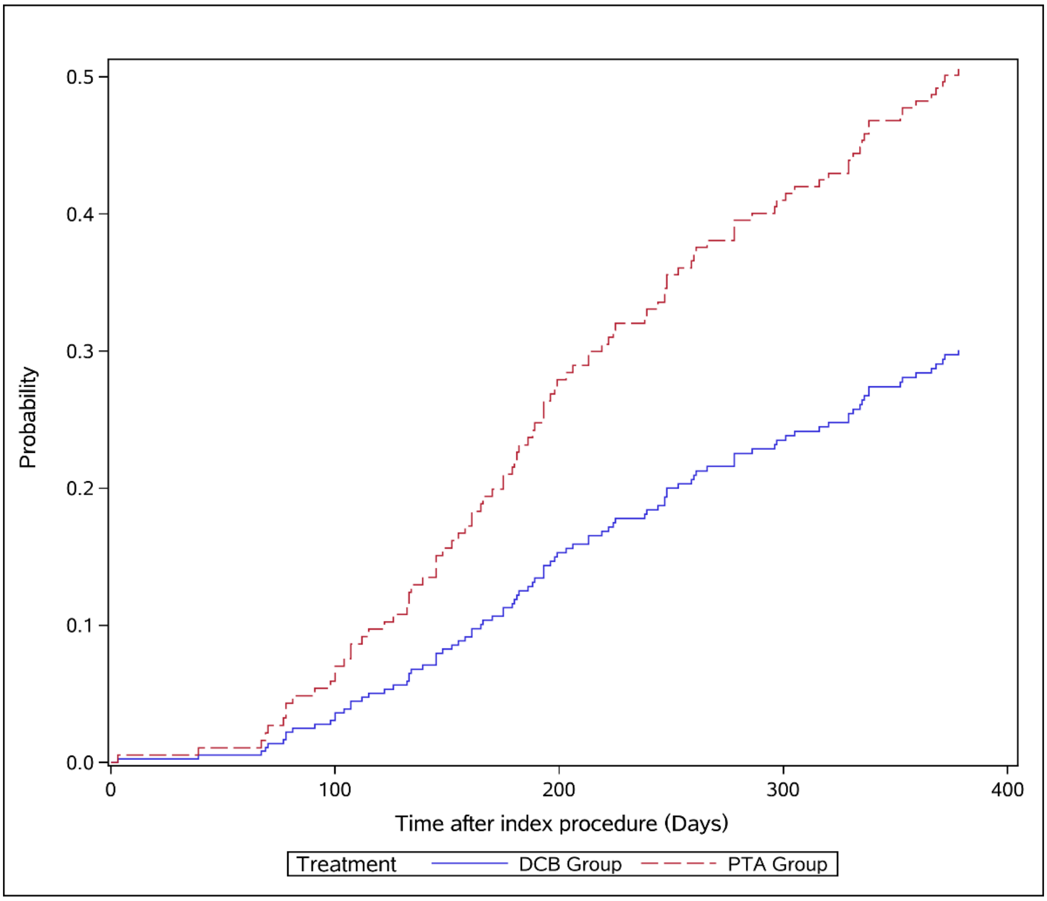

Supplemental Figure 1. Cumulative incidence functions of reintervention risk during 12 months. The difference of reintervention risk between drug-coated balloon (DCB) group and PTA group (Percutaneous Transluminal Angioplasty using plain old balloon) was significant (Log rank, P<0.001).
